# High-quality bulk hybrid perovskite single crystals within minutes by inverse temperature crystallization

**DOI:** 10.1038/ncomms8586

**Published:** 2015-07-06

**Authors:** Makhsud I. Saidaminov, Ahmed L. Abdelhady, Banavoth Murali, Erkki Alarousu, Victor M. Burlakov, Wei Peng, Ibrahim Dursun, Lingfei Wang, Yao He, Giacomo Maculan, Alain Goriely, Tom Wu, Omar F. Mohammed, Osman M. Bakr

**Affiliations:** 1 Division of Physical Sciences and Engineering, Solar and Photovoltaics Engineering Research Center, King Abdullah University of Science and Technology (KAUST), Thuwal 23955-6900, Kingdom of Saudi Arabia; 2 Department of Chemistry, Faculty of Science, Mansoura University, Mansoura 35516, Egypt; 3 Mathematical Institute, University of Oxford, Woodstock Road, Oxford OX2 6GG, UK; 4 Materials Science and Engineering, King Abdullah University of Science and Technology (KAUST), Thuwal 23955-6900, Kingdom of Saudi Arabia; 5 Imaging and Characterization Lab, King Abdullah University of Science and Technology (KAUST), Thuwal 23955-6900, Kingdom of Saudi Arabia

## Abstract

Single crystals of methylammonium lead trihalide perovskites (MAPbX_3_; MA=CH_3_NH_3_^+^, X=Br^−^ or I^−^) have shown remarkably low trap density and charge transport properties; however, growth of such high-quality semiconductors is a time-consuming process. Here we present a rapid crystal growth process to obtain MAPbX_3_ single crystals, an order of magnitude faster than previous reports. The process is based on our observation of the substantial decrease of MAPbX_3_ solubility, in certain solvents, at elevated temperatures. The crystals can be both size- and shape-controlled by manipulating the different crystallization parameters. Despite the rapidity of the method, the grown crystals exhibit transport properties and trap densities comparable to the highest quality MAPbX_3_ reported to date. The phenomenon of inverse or retrograde solubility and its correlated inverse temperature crystallization strategy present a major step forward for advancing the field on perovskite crystallization.

## Abstract (web summary)

Hybrid perovskites are a promising class of materials for photovoltaic applications. Here, addressing the need for high-quality hybrid perovskite materials, the authors achieve the rapid growth of hybrid perovskite single crystals of high quality by inverse temperature crystallization.

## Graphical abstract

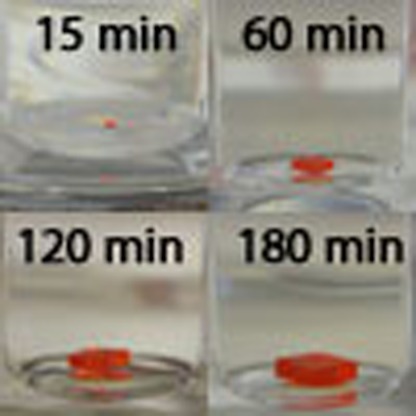

Organo-lead trihalide hybrid perovskites (MAPbX_3_; MA=CH_3_NH_3_^+^, X=Br^−^ or I^−^) have been widely investigated for solar cells^1^,^2^,^3^,^4^,^5^,^6^,^7^,^8^, lasing^9^, light-emitting diodes^10^, photodetectors^11^ and hydrogen production^12^. This considerable interest in organo-lead trihalide perovskites is because of their tunable optical properties, high-absorption coefficients, long-ranged balanced electron and hole transport^13^, low cost and facile deposition techniques^14^,^15^,^16^. In particular, single crystals of MAPbBr_3_ and MAPbI_3_ were shown to possess long carrier diffusion lengths and a remarkably low trap-state densities, which is comparable to the best photovoltaic-quality silicon^17^. These properties provide a view of the ultimate potential of hybrid perovskites, and make single crystals of MAPbX_3_ a highly desirable semiconductor for optoelectronic applications that are much broader than their polycrystalline thin film counterpart. However, the reported solution crystallization processes for perovskite single crystals suffer from very slow growth rates and no shape control over the resultant crystals^17^,^18^,^19^,^20^,^21^. The highest reported growth rate was estimated to be ∼26 mm^3^ per day (∼1 mm^3^ h^−1^), based on a MAPbI_3_ crystal with the dimensions of 10 mm × 10 mm × 8 mm that took a month to grow^20^. A radically faster crystallization technique that could also address the need for a diverse variety of crystal geometries will allow more extensive use of hybrid perovskite single crystals.

The choice of a suitable solvent medium has always been a defining factor for the quality of the ensuing crystals. In the case of hybrid perovskites, the most widely used solvents are *γ*-butyrolactone (GBL), *N,N*-dimethylformamide (DMF) and dimethylsulphoxide (DMSO). The solubility of PbX_2_ and MAX in these solvents or their mixtures was found to vary; hence, it was previously reported that MAPbBr_3_ crystallized more aptly from DMF while MAPbI_3_ crystallized better from GBL^17^. Perovskite crystallization from aqueous solution was also reported, in which crystals were formed, classically, on cooling a preheated solution^20^. It is generally the norm that solutes tend to have a higher degree of solubility at higher temperatures; hence, a good solvent for crystallization will dissolve more precursors when hot, while cooling down induces supersaturation commencing the crystallization. On the other hand, a decrease of solute solubility in solvents with increasing temperature (that is, inverse temperature or retrograde solubility) is a rare occurrence, that is, only displayed by a few materials^22^.

Here we show that MAPbX_3_ perovskites exhibit inverse temperature solubility behaviour in certain solvents. This novel phenomenon in hybrid perovskites enabled us to design an innovative crystallization method for these materials, referred to here as inverse temperature crystallization (ITC), to rapidly grow high-quality size- and shape-controlled single crystals of both MAPbBr_3_ and MAPbI_3_, at a rate that is an order of magnitude faster than the previously reported growth methods^17^,^18^,^19^,^20^,^21^. The versatility of our approach provides the continuous enlargement of crystals, through replacement of the depleted growth solution, and the use of templates for controlling their shapes.

## Results

### Single crystal growth and structural characterization

We noticed the rapid formation of small MAPbBr_3_ perovskite precipitates at high temperatures in some concentrated solutions (for example, DMF) and not in others (for example, DMSO). On the other hand, GBL could not be used as a solvent because of the very low solubility of MAPbBr_3_ (<0.05 g ml^−1^ at both room temperature and 80 °C). The effect of the different solvents could be related to their varying degrees of coordination with the precursors, as it was previously reported that DMSO may retard the crystallization process because of its strong binding to the lead precursor^23^,^24^,^25^.

**Figure 1:**
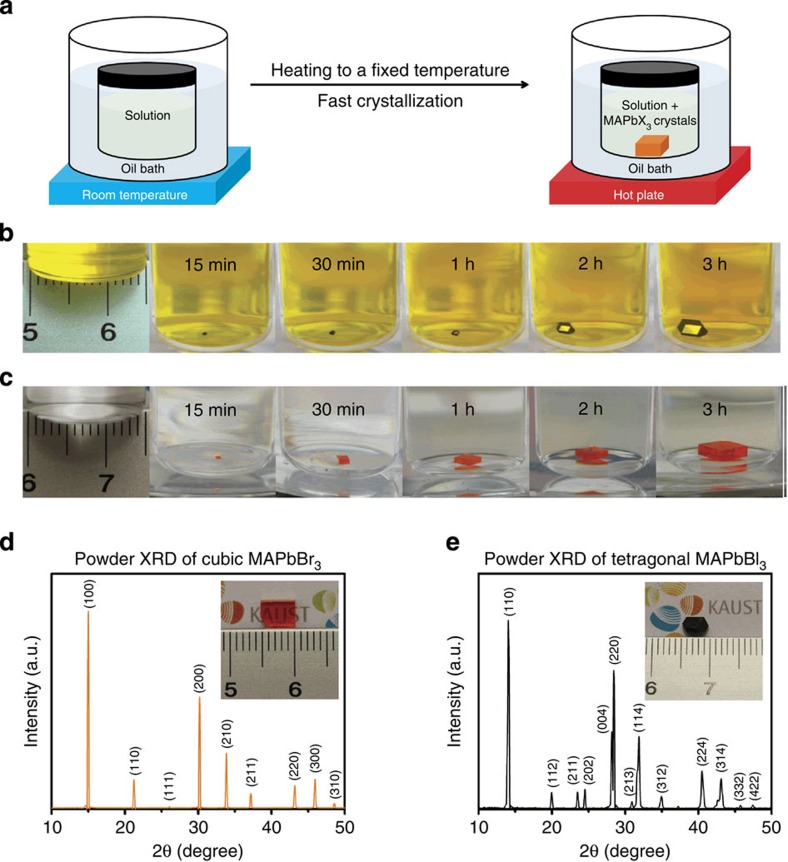
Crystal growth process and powder X-ray diffraction. (**a**) Schematic representation of the ITC apparatus in which the crystallization vial is immersed within a heating bath. The solution is heated from room temperature and kept at an elevated temperature (80 °C for MAPbBr_3_ and 110 °C for MAPbI_3_) to initiate the crystallization. (**b**,**c**) MAPbI_3_ and MAPbBr_3_ crystal growth at different time intervals. (**d**,**e**) Powder X-ray diffraction of ground MAPbBr_3_ and MAPbI_3_ crystals. Insets: pictures of the corresponding crystals grown within a non-constraining vessel geometry.

Consequently, DMF was chosen for MAPbBr_3_ ITC. Through studying the solubility of MAPbBr_3_ in DMF, we observed that it drops markedly from 0.80±0.05 g ml^−1^ at room temperature to 0.30±0.05 g ml^−1^ at 80 °C. This inverse solubility phenomenon was used to crystallize MAPbX_3_ rapidly in hot solutions as illustrated in Fig. 1a. Through balancing both temperature and concentration of precursors in DMF, only a few crystals were formed. For instance, by setting the temperature of the heating bath at 80 °C usually <5 crystals are formed in case of 1 M solution of PbBr_2_ and MABr (Supplementary Fig. 1). Inspired by this observation, we repeated the same procedure and studied different solvents that could lead to the same effect in MAPbI_3_. Unlike MAPbBr_3_, ITC of MAPbI_3_ was only possible in GBL solution, while no precipitates were observed in the case of DMF or DMSO.

As expected, we observed that the crystallization process in both MAPbBr_3_ and MAPbI_3_ is reversible and the crystals dissolve back when cooled to room temperature. It is also worth mentioning that individual precursors PbX_2_ and MAX did not show any inverse solubility behaviour (that is, saturated solutions of the individual precursor did not show precipitation on heating), implying that the phenomenon is tied to the perovskite structure.

The growth process of MAPbI_3_ crystal using the ITC technique was recorded on video using time-accelerated mode (Supplementary Movie 1), several snapshots of which are shown in Fig. 1b. Individual MAPbI_3_ crystal was calculated to grow at a rate of ∼3 mm^3^ h^−1^ for the first hour, a rate that significantly increases to ∼9 mm^3^ h^−1^ for the second hour and to ∼20 mm^3^ h^−1^ for the following hour. This value is an order of magnitude greater than the previously reported highest growth rate^20^. An even faster growth rate was observed for MAPbBr_3_ crystals, reaching up to 38 mm^3^ h^−1^ for the third hour (Fig. 1c), resulting in a higher yield in comparison with MAPbI_3_. Powder X-ray diffraction patterns of the ground crystals demonstrate pure perovskite phase for both MAPbBr_3_ and MAPbI_3_ (Fig. 1d,e). Single-crystal X-ray diffraction analysis showed a good match with previous single crystals grown at room temperature using antisolvent vapour-assisted crystallization^17^ (Supplementary Table 1). Scanning electron microscopy images of the cleaved crystals show the absence of any grain boundaries, indicating the single-crystalline nature of both crystals (Supplementary Fig. 2).

**Figure 2:**
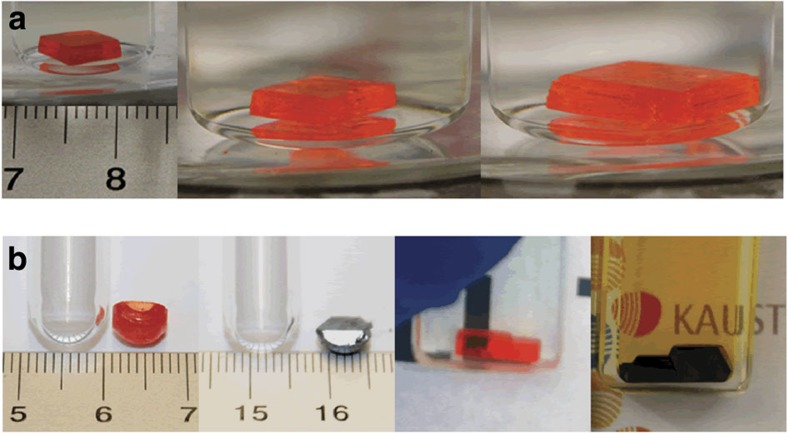
Continuous growth and crystal shape control. (**a**) Continuous growth of an MAPbBr_3_ crystal by moving the crystal into a larger vial with a fresh growth solution. (**b**) Shape-controlled crystals of MAPbBr_3_ (orange) and MAPbI_3_ (black) by varying the geometry of the confining vessel. From left to right—crystals grown in a round-bottom test tube and a 2-mm cuvette.

Further growth of the crystal was achieved by carefully removing the crystal and placing it in a fresh 1 M solution of the precursors (Fig. 2a). As shown in Supplementary Movie 2 for MAPbBr_3_, we observed a possible shape control of the crystal by the geometry of crystallization vessel. Hence, single crystals of MAPbBr_3_ and MAPbI_3_ were synthesized with a number of different shapes by changing the geometry of the vessel in which crystallization takes place (Fig. 2b).

### Optical and transport properties

**Figure 3:**
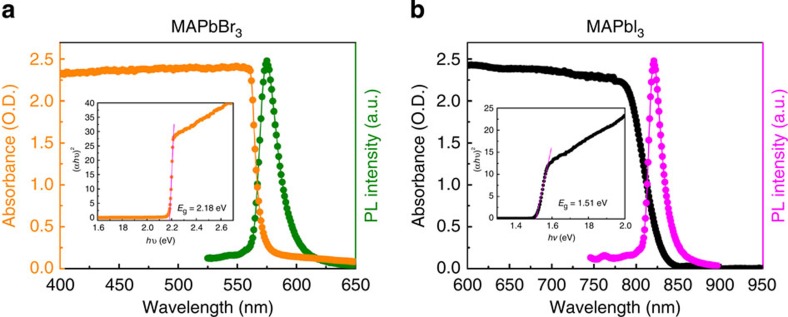
Steady-state absorption and photoluminescence. (**a**) MAPbBr_3_ single crystal. (**b**) MAPbI_3_ single crystal. Insets: corresponding Tauc plots displaying the extrapolated optical band gaps.

Further, we investigated optical and transport properties of the crystals, demonstrating that MAPbX_3_ obtained using ITC in few hours are comparable quality to previously reported crystals grown in several weeks. From the steady-state absorption measurements a sharp band edge is observed (Fig. 3a,b). Band gaps extracted from Tauc plots show values of 2.18 and 1.51 eV for MAPbBr_3_ and MAPbI_3_, respectively. The band gap values for crystals grown using ITC are in a good agreement with the values reported for single crystals grown at room temperature through antisolvent vapour-assisted crystallization^17^. The photoluminescence peak position of MAPbBr_3_ and MAPbI_3_ single crystals is located at 574 and 820 nm, respectively, matching the values reported earlier for the same single crystals grown using antisolvent vapour-assisted crystallization^17^.

To investigate the excited-state lifetime of these single crystals, we monitored both the ground-state bleach recovery and the excited-state absorption in the nano- and microsecond time regime using nanosecond transient absorption spectroscopy with broadband capabilities. Two time components are observed for both single crystals. A fast component of *τ*≈28±5 ns and *τ*≈18±6 ns together with a slower decay of *τ*≈300±26 ns and *τ*≈570±69 ns were measured for MAPbBr_3_ and MAPbI_3_ crystals, respectively. These measured surface (fast component) and bulk (slow component) carrier lifetimes are in good agreement with the ones reported recently for the same kinds of single crystals^17^.

**Figure 4:**
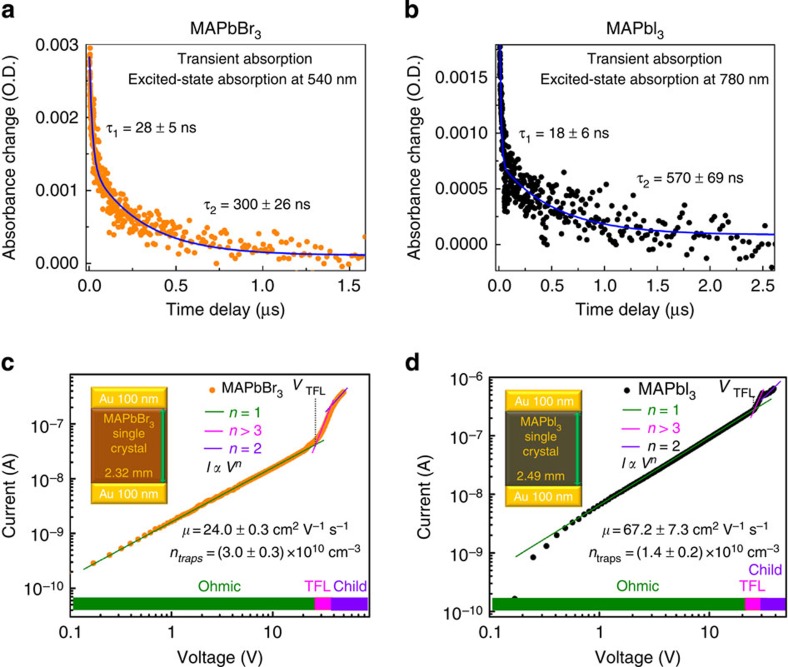
Carrier lifetime measurements and *I*–*V* traces. (**a**,**b**) Transient absorption of (**a**) MAPbBr_3_ and (**b**) MAPbI_3_ crystals. (**c**,**d**) *I–V* of perovskite crystals exhibiting different regions obtained from the log *I* versus log *V* plots. The regions are marked for Ohmic (*IαV*^*n*=1^), TFL (*IαV*^*n*>3^) and Child's regime (*IαV*^*n*=2^). The trap densities were calculated from the Child's regime shown in (**c**,**d**).

The carrier mobility *μ* (*μ*=*μ*_p_≈*μ*_n_, where *μ*_p_ and *μ*_n_ are hole and electron mobility, respectively, as MAPbX_3_ is an intrinsic semiconductor)^26^ of MAPbX_3_ (X=Br^−^, I^−^) was estimated from the dark current*–*voltage (*I–V)*characteristics, following the standard space charge-limited current model. The *I–V* traces showed the Mott–Gurney's power law dependence, for instance, an Ohmic region at the lower and a space charge-limited current model at higher bias. A quadratic dependence of the transition from the Ohmic to Child's law through the trap filled limit (TFL) was observed in both MAPbBr_3_ and MAPbI_3_ crystals. The carrier mobilities and the trap densities (*n*_traps_) were estimated to be 24.0 cm^2^ V^−1^ s^−1^ and 3 × 10^10^ cm^−3^ for MAPbBr_3_ crystals (Fig. 4c), as well as 67.2 cm^2^ V^−1^ s^−1^ and 1.4 × 10^10^ cm^−3^ for MAPbI_3_ crystals (Fig. 4d).

We calculated the carrier diffusion length by combining carrier lifetime with mobility *L*_D_=(*μτk*_B_*T*/*e*)^1/2^ (where *k*_B_ is Boltzmann's constant and *T* is the sample's temperature). By using the longer carrier lifetime (bulk component), a best-case carrier diffusion length was calculated to be ∼4.3 μm for MAPbBr_3_ and ∼10.0 μm for MAPbI_3_. A worst-case diffusion length could be estimated from the shorter carrier lifetime, corresponding to the surface component: ∼1.3 and ∼1.8 μm for MAPbBr_3_ and MAPbI_3_, respectively. Hence, despite the rapid rate with which crystals were grown via ITC, their transport characteristics together with trap-state densities are comparable to single crystals prepared with classical techniques, which were grown over a much longer period of time.

## Discussion

We observed experimentally that perovskite crystals formed in the precursor solution at elevated temperatures dissolved back when the solution temperature was decreased to room temperature. This observation demonstrates that the thermodynamic stability of a precipitated hybrid perovskite compound has seemingly paradoxical temperature dependence, since simple monomolecular compounds are expected to dissolve at higher temperatures. Therefore, it is instructive to analyse how such a situation may arise. We hypothesize that this phenomenon might be related to the formation of complexes of precursors (whose nature is not the subject of this report and is under intensive study) or their products with the solvent^23^,^27^,^28^. The theory presented below illustrates how these complexes can affect the temperature-dependent stability of the precipitate and reverse its effect depending on the different parameters of the system.

Suppose there is only one type of molecular precursor controlling the crystallization of a complex compound such as a perovskite, then the formation of complexes involving the precursor molecule and solvent molecules may significantly affect the precipitation of the compound. To illustrate this situation we analyse the thermodynamic stability of a monomolecular precipitate made of molecules A in the solution, where molecules A can form complexes with solvent molecules, the complex's binding energy being *ɛ*_C_. As an ultimate stable state always contains only one precipitated particle, and to avoid secondary issues related to crystal facets, we assume that a single precipitated A-particle is placed in a unit volume of solution and has a spherical shape.

In general, the stability of precipitated solids in a solution is determined by several conditions based on the balance of chemical potentials of all its molecular or atomic constituents present in the solution and in the solid form. These conditions must also take into account the presence of complexes formed by the constituents in the solution. In our case, these conditions are the equality of the chemical potentials of A-molecules in the particle and solution (that is, *μ*_P_=*μ*_A_), and of the complex's chemical potential, *μ*_C_, and the sum of chemical potentials for all complex constituents (one A-molecule and *j* solvent molecules); that is, *μ*_C_=*jμ*_S_+*μ*_A_. Expressed in terms of concentrations and binding energies, these conditions read (Supplementary Note 1 for details):


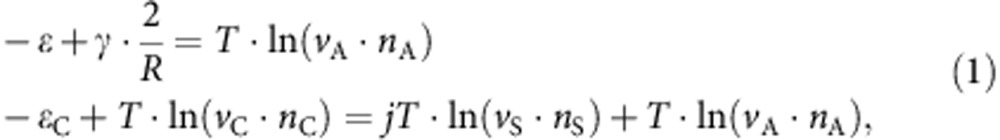


where *ɛ* is the cohesive energy of A-molecule in the particle, *γ* is the surface energy per A-molecule, *R* is the particle radius measured in terms of the characteristic intermolecular distance, *T* is the solution temperature, *n*_C_ and *n*_A_ are the number concentrations in the solution; and *v*_C_, *v*_A_ and *v*_S_ are the characteristic volumes of the complex, A-molecule and solvent molecule, respectively. Resolving equation (1) with respect to concentrations gives:


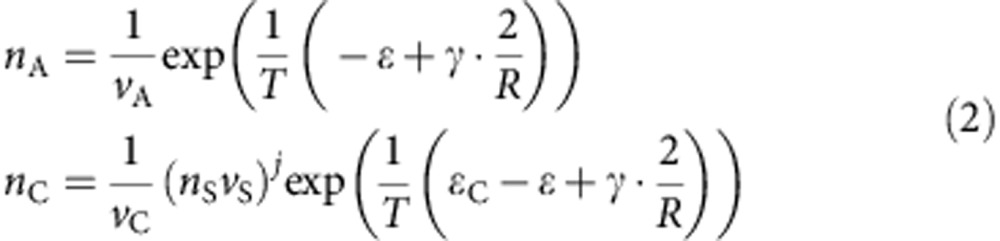


To simplify the analysis we consider the limit when the particle size is large enough, that is, far from its critical value. In that case, the surface energy contributions in the exponents of equation (2) can be neglected, that is, we take the limit *γ*→0. The total number concentration *m*_C_ of all A-molecules consists of the part *n*_P_ forming the particle, the part *n*_C_ forming complexes and the part *n*_A_ of individual molecules in the solution such that *m*_A_=*n*_P_+*n*_C_+*n*_A_. Using these constraints and equation (2) we obtain for the number fraction of precipitated A-molecules, *n*_p_*v*_*A*_:


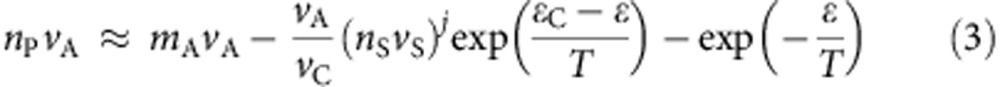


To illustrate the effect of temperature on *n*_P_, it is convenient to analyse the derivative of equation (3):


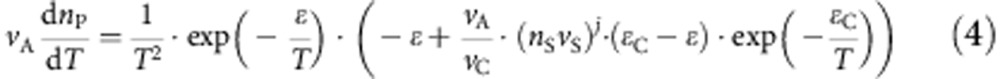


If d*n*_P_/d*T*<0 then it would mean that the precipitated mass (that is, A-particle size) decreases with increasing temperature—the situation typically observed for most materials precipitating from solution. In contrast, if d*n*_P_/d*T*>0 an interesting situation occurs in which an increase in temperature results in an increase in the precipitated number of A-molecules, as observed experimentally for hybrid perovskites. This effect, as can be seen from equation (4), takes place if


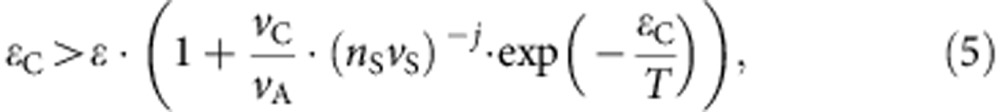


or if we accept that 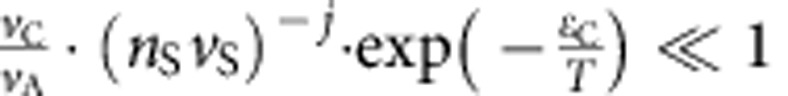 (that is, large enough *ɛ*_C_/*T* ratio), then the inequality given by equation (5) reduces to *ɛ*_C_>*ɛ*. These analytical relation can be further understood in physical terms as follows: at low temperatures most of the A-molecules are bound in the complexes with the solvent; therefore, the solution has no supersaturation in terms of concentration of unbound A-molecules. When the temperature increases, the concentration of unbound A-molecules increases (because of dissociation of the complexes) and may reach the supersaturation, thus triggering the precipitation of A-particles. Conversely, when the temperature of the solution containing the precipitated A-particle is decreased, the concentration *n*_A_ of unbound A-molecules is also decreased because of formation of many more complexes with solvent. This decrease in *n*_A_ makes the solution too diluted in A-molecules such that the particle has to transfer some molecules to the solution, that is, it dissolves. It should be noted that the process of crystallization is endothermic with respect to A-molecules, as a molecule moves from the complex with higher binding energy to the precipitate, where its binding (cohesive) energy is lower. Therefore, the crystallization reaction consumes thermal energy.

The temperature behaviour described by equation (5) provides a qualitative framework to explain the effects observed experimentally for perovskite materials. A quantitative analysis requires a detailed investigation of the molecular content of the precursor solution, a subject of future research.

In summary, we report the novel observation of inverse solubility of hybrid organo-lead trihalide perovskites. A careful choice of solvent, temperature and other parameters made it possible to utilize this method to rapidly grow single crystals of MAPbBr_3_ and MAPbI_3_ in hot solutions via ITC. Despite the fact that these crystals grow very fast, they exhibit carrier transport properties comparable to those grown by the usual cooling or antisolvent vapour-assisted crystallization techniques. The ‘quantum leap' in crystal growth rates in ITC, over the previously reported growth methods so far used for single crystal-hybrid perovskites, represents a major breakthrough in the field of perovskite single crystals for enabling the wide applications of these remarkable semiconductor materials.

## Methods

### Chemicals and reagents

Lead bromide (≥98%), lead iodide (99.999% trace metal basis), DMF (anhydrous, 99.8%) and GBL (≥99%) were purchased from Sigma Aldrich. MABr and MAI were purchased from Dyesol Limited (Australia). All salts and solvents were used as received without any further purification.

### Synthesis of MAPbX_3_ single crystals

One molar solution containing PbX_2_ and MAX was prepared in DMF or GBL for X=Br^−^, I^−^, respectively. The bromide solution was prepared at room temperature, whereas the iodide solution was heated up to 60 °C. The solutions were filtered using PTFE filter with 0.2-μm pore size. Two millilitres of the filtrate were placed in a vial and the vial was kept in an oil bath undisturbed at 80 and 110 °C for Br- and I-based perovskites, respectively. All procedures were carried out under ambient conditions and humidity of 55–57%. The crystals used for measurements were grown for 3 h. The reaction yield for MAPbBr_3_ and MAPbI_3_ was calculated to be 35 and 11 wt %, respectively.

### Measurement and characterization

Powder X-ray diffraction was performed on a Bruker AXS D8 diffractometer using Cu-Kα radiation. Single-crystal X-ray diffraction was performed on Bruker D8 Venture, CMOS detector, microfocus copper source. The steady-state absorption and photoluminescence were recorded using Cary 6000i spectrophotometer with an integrating sphere and Edinburgh Instrument spectrofluorometer, respectively. Time-resolved transient absorption decays were measured with a femto-nanoseconds pump−probe set-up. The excitation pulse at 480 nm was generated using a spectrally tunable optical Parametric Amplifier (Light Conversion LTD) integrated to a Ti:sapphire femtosecond regenerative amplifier operating at 800 nm with 35 fs pulses and a repetition rate of 1 kHz. The white light probe pulse, on the other hand, was generated by a super continuum source^29^,^30^. The pump and probe beams were overlapped spatially and temporally on the sample, and the transmitted probe light from the samples was collected and focused on the broad-band ultraviolet–visible-near-infrared detectors to record the time-resolved excitation-induced difference spectra. *I–V* characteristics were carried out in the dark under vacuum (∼10^−4^ mbar) at 300 K, in the simple two electrode configuration (Au/MAPbX_3_/Au). The perovskite crystal was sandwiched between the rectangular electrodes (3 mm × 2 mm) Au (100 nm), deposited on both sides of the single crystal, by an Angstrom thermal evaporator at a 0.5 Å s^−1^ deposition rate. The thickness and rate of deposition during the evaporation of Au contact was monitored by an Inficon thickness monitor. The thickness of MAPbBr_3_ and MAPbI_3_ crystals were measured as 2.32 and 2.49 mm, respectively, by the digital Vernier caliper. The typical nonlinear dark current, voltage plots followed the Lampert's theory, where the current was found to be limited by the trap-assisted space charge conduction. Onset voltage (*V*_TFL_) for the TFL was used for the calculation (equation (6)) of density of traps (*n*_traps_) in the perovskite crystals


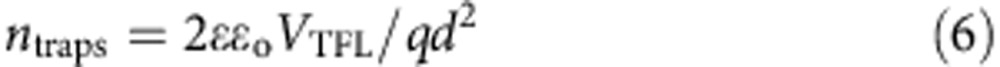


where *q* is the electronic charge, *d* is the thickness of the crystal, *ɛ* is the dielectric constant of the material (25.5 for MAPbBr_3_ and 32 for MAPbI_3_)^19^,^31^ and *ɛ*_o_ is the vacuum permittivity.

## Additional information

**How to cite this article:** Saidaminov, M. I. *et al*. High-quality bulk hybrid perovskite single crystals within minutes by inverse temperature crystallization. *Nat. Commun.* 6:7586 doi: 10.1038/ncomms8586 (2015).

## Supplementary Material

Supplementary Figures, Supplementary Table, Supplementary Note and Supplementary References Supplementary Figures 1-2, Supplementary Table 1, Supplementary Note 1 and Supplementary References

Supplementary Movie 1 Time-accelerated video of MAPbI_3_ crystal growth from 1 M solution of MAPbI_3_ in GBL by Inverse Temperature Crystallization at 110 °C.

Supplementary Movie 2 Time-accelerated video of MAPbBr_3_ crystal growth from 1 M solution of MAPbBr_3_ in DMF by Inverse Temperature Crystallization at 80 °C
