# Supplementary material for: High-quality bulk hybrid perovskite single crystals within minutes by inverse temperature crystallization
Source: Nat Commun. 2015 Jul 6;6:7586. doi: 10.1038/ncomms8586 (PMC4544059; doi:10.1038/ncomms8586)
Supplement: Supplementary Figures, Supplementary Table, Supplementary Note and Supplementary References — Supplementary Figures 1-2, Supplementary Table 1, Supplementary Note 1 and Supplementary References [file ncomms8586-s1.pdf]

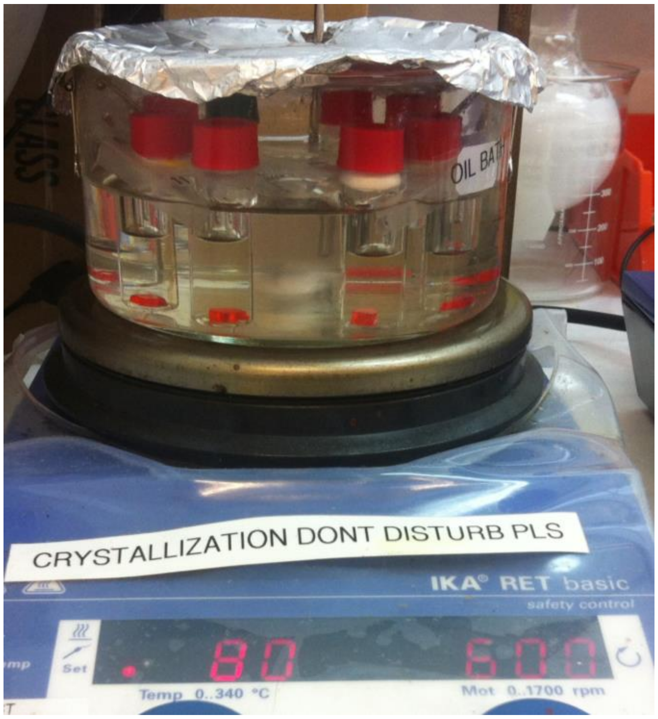

Supplementary Figure 1. Picture of crystallization setup. This picture demonstrates, that all 8 vessels containing 1 M $MAPbBr_3$ in DMF produced less than 5 crystals, in particular, 3 of them produced an individual $MAPbBr_3$ crystal. Time-accelerated video of one of these individual crystals growth is presented in Supplementary Movie 2.

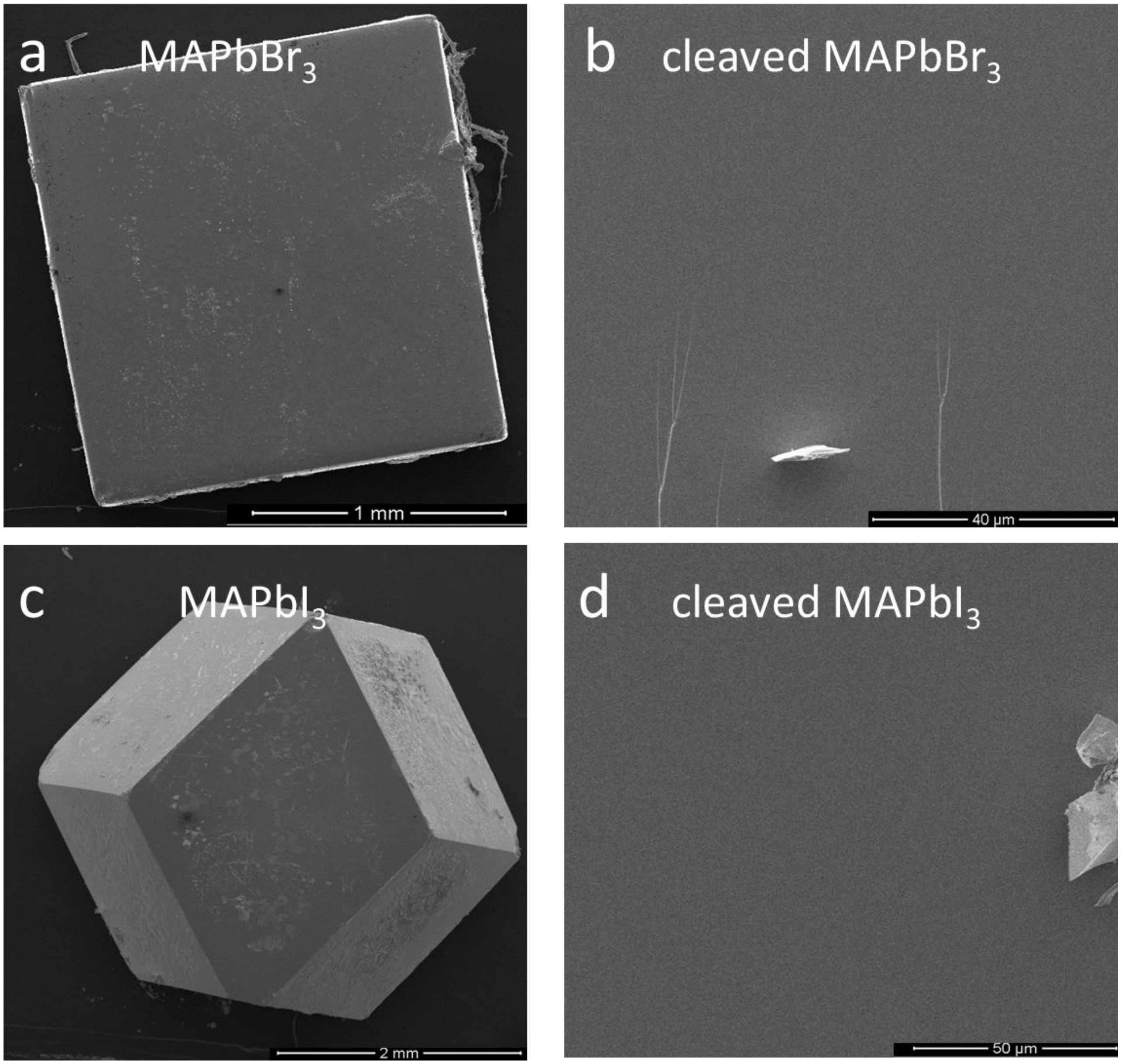

Supplementary Figure 2. SEM images of the surface and cleaved crystals. SEM of (a) surface of $MAPbBr_3$, (b) cleaved $MAPbBr_3$, (c) surface of $MAPbI_3$ and (d) cleaved $MAPbI_3$. Excess precursors from solution can be observed on the surface of the crystals. No grain boundaries are found on the surface or core of cleaved crystals indicating the single crystalline nature of both crystals. The dusts in (b,d) indicate the good focus of electron beam.

Supplementary Table 1. Single crystal XRD data.

**Compound**	**$MAPbBr_3$**	**$MAPbI_3$**
$D_{calc.}$/ g cm^{-3}	3.582	3.947
μ/mm^{-1}	55.601	105.893
Max Size/mm	0.98	0.58
Mid Size/mm	0.09	0.56
Min Size/mm	0.05	0.53
T/K	250(2)	250(2)
Crystal System	cubic	tetragonal
Space Group	Pm3m	I4/m
a/Å	5.9173(6)	8.8310(5)
b/Å	5.9173(6)	8.8310(5)
c/Å	5.9173(6)	12.6855(7)
$\alpha/^{\circ}$	90	90
$\beta/^{\circ}$	90	90
$\gamma/^{\circ}$	90	90
V/Å^3	207.19(6)	989.30(12)
Z	1	4
Z'	0.02083	0.125
$\Theta_{min}/^{\circ}$	10.617	6.981
$\Theta_{max}/^{\circ}$	71.520	72.151
Measured Refl.	1572	2093
Independent Refl.	64	278
Reflections Used	64	274
R_{int}	0.0659	0.0971
Parameters	4	9
Restraints	0	0
Largest Peak	4.507	8.090
Deepest Hole	-6.787	-17.741
GooF	1.336	1.491
wR_2 (all data)	0.2197	0.3021
wR_2	0.2197	0.3010
R_1 (all data)	0.0868	0.1034
R_1	0.0868	0.1029

Single crystals of both materials have been obtained and their unit cells have been verified by single crystal X-ray diffraction. The unit cell dimensions as well as the space groups (cubic, space group Pm3m, a = 5.9173(6) Å for $MAPbBr_3$ and tetragonal, space group I4/m, a = b = 8.8310(5) Å, c = 12.6855(7) Å for $MAPbBr_3$) are in excellent agreement with literature reports[1-3].

Supplementary Note 1

The free energy of a system of unit volume containing a number n_S of solvent molecules in the solution and numbers n_A and n_C of, respectively, isolated A-molecules, and the complexes is

$$\begin{aligned} G = &-\varepsilon n_P + \gamma \cdot S_P - \varepsilon_C \cdot n_C + T n_C \cdot \ln\left(n_C v_C\right) + T n_A \cdot \ln\left(n_A v_A\right) + T n_S \cdot \ln\left(n_S v_S\right) \\ &- T \cdot \left(n_A + n_S + n_C\right) \cdot \ln\left(n_A v_A + n_S v_S + n_C v_C\right) \end{aligned}, \quad (1)$$

where ε is the cohesive energy of A-molecules in the particle, γ is the surface energy per molecule, ε_C is the binding energy of the complexes, S_P is the number of A-molecules on the surfaces of A-particle, T is the solution temperature and v_S, v_C and v_A are the characteristic volumes of the solvent molecule, the complex, and the A-molecule, respectively. It is convenient to introduce total concentrations of A- and solvent molecules $m_A = n_A + n_P + n_C$ and $m_S = n_S + j \cdot n_C$, respectively, where j is the number of solvent molecules in the A-solvent complex. Expressing n_S and n_A in terms of the total concentrations allows rewriting equation (1) in the form

$$\begin{aligned} G = &-\varepsilon n_P + \gamma \cdot S_P - \varepsilon_C \cdot n_C + T n_C \cdot \ln\left(n_C v_C\right) + T\left(m_A - n_P - n_C\right) \cdot \ln\left(\left(m_A - n_P - n_C\right) v_A\right) \\ &+ T\left(m_S - j n_C\right) \cdot \ln\left(\left(m_S - j n_C\right) v_S\right) \\ &- T\left(m_A + m_S - n_P - j n_C\right) \cdot \ln\left(v_A m_A + v_S m_S - v_A n_P + n_C \cdot \left(v_C - v_A - j v_S\right)\right) \end{aligned} \quad (2)$$

We are interested in both the equilibrium size of A-particles and the concentration n_C of A complexes with solvent. Therefore we choose n_P and n_C as independent variables. Minimization of the free energy with respect to these variables leads to the equations

$$\frac{\partial G}{\partial n_\mathrm{P}} = -\varepsilon + \gamma \cdot \frac{2}{R} - T \cdot \ln\left(n_\mathrm{A} v_\mathrm{A}\right) = 0$$
$$\frac{\partial G}{\partial n_\mathrm{C}} = -\varepsilon_\mathrm{C} + T \cdot \ln\left(n_\mathrm{C} v_\mathrm{C}\right) - T \cdot \ln\left(n_\mathrm{A} v_\mathrm{A}\right) - jT \cdot \ln\left(n_\mathrm{S} v_\mathrm{S}\right) = 0 \quad (3)$$

The first line gives an equality of the chemical potentials of A-molecules in the particle $\mu_\mathrm{AP} = -\varepsilon + 2\gamma / R$ and in the solution $\mu_\mathrm{AS} = T \cdot \ln\left(n_\mathrm{A} v_\mathrm{A}\right)$. The second line states that chemical potential of the complex is equal to the sum of chemical potentials of A-molecule and j solvent molecules involved in complex formation $\mu_\mathrm{SS} = T \cdot \ln\left(n_\mathrm{S} v_\mathrm{S}\right)$. Solving the equations

$$-\varepsilon + \gamma \cdot \frac{2}{R} = T \cdot \ln\left(n_\mathrm{A} v_\mathrm{A}\right)$$
$$-\varepsilon_\mathrm{C} + T \cdot \ln\left(n_\mathrm{C} v_\mathrm{C}\right) = T \cdot \ln\left(n_\mathrm{A} v_\mathrm{A}\right) + jT \cdot \ln\left(n_\mathrm{S} v_\mathrm{S}\right) \quad (4)$$

with respect to n_A and n_C gives

$$n_\mathrm{A} = \frac{1}{v_\mathrm{A}} \exp\left(\frac{1}{T}\left(-\varepsilon + \gamma \cdot \frac{2}{R}\right)\right)$$
$$n_\mathrm{C} = \frac{1}{v_\mathrm{C}} \left(n_\mathrm{S} v_\mathrm{S}\right)^j \exp\left(\frac{1}{T}\left(\varepsilon_\mathrm{C} - \varepsilon + \gamma \cdot \frac{2}{R}\right)\right) \quad (5)$$

These equations contain the particle radius R, which can be expressed in terms of n_P. Therefore, these equations can be solved numerically. However, we can understand qualitatively the temperature effect on n_P by considering the particle size to be far from its critical value (where the surface energy is really important) when we may set $\gamma \approx 0$. In that limit the number fraction of precipitated molecules is

$$n_\mathrm{P} v_\mathrm{A} = m_\mathrm{A} v_\mathrm{A} - \frac{v_\mathrm{A}}{v_\mathrm{C}} \left(n_\mathrm{S} v_\mathrm{S}\right)^j \exp\left(\frac{\left(\varepsilon_\mathrm{C} - \varepsilon\right)}{T}\right) - \exp\left(-\frac{\varepsilon}{T}\right). \quad (6)$$

Supplementary References

1. Shi D, *et al.* Low trap-state density and long carrier diffusion in organolead trihalide perovskite single crystals. *Science* **347**, 519-522 (2015).

2. Dong Q, *et al.* Electron-hole diffusion lengths > 175 μm in solution-grown $CH_3NH_3PbI_3$ single crystals. *Science* **347**, 967-970 (2015).

3. Baikie T, *et al.* Synthesis and crystal chemistry of the hybrid perovskite (CH_3NH_3) PbI_3 for solid-state sensitised solar cell applications. *J. Mater. Chem. A* **1**, 5628-5641 (2013).
